# Variable Clinical Presentations of Riedel's Thyroiditis: Report of Two Cases

**DOI:** 10.1155/2011/709264

**Published:** 2011-08-08

**Authors:** Hamidreza Zakeri, Zahra Kashi

**Affiliations:** ^1^Tehran University of Medical Sciences, P.O. Box 13185-1678, Tehran, Iran; ^2^Mazandaran University of Medical Sciences, Sari, Iran

## Abstract

Riedel's thyroiditis is a rare inflammatory disease of the thyroid gland and has been reported in 0.05% of thyroid surgeries. Herein we report two cases of Riedel's thyroiditis with variable clinical presentations. One of these cases was a 51-year-old man who was presented with hypothyroidism and the other a 17-year-old young male with thyrotoxicosis. In these cases, age may be a determining factor in presenting symptoms and signs, disease process, and response to treatment.

## 1. Introduction

Riedel's (fibrosing) thyroiditis is a rare inflammatory disease of the thyroid gland and has been reported in 0.05% of thyroid surgeries. It was first described by Bernhard Riedel in 1896 [1] as a fibroinflammatory process associated with thyroid infiltratration. 

The etiology of Riedel's thyroiditis is unknown, although the autoimmune process is the most probable etiology [2, 3]. It causes extensive fibrosis in thyroid tissue which involves surrounding tissues and causes swelling, dysphagia, and voice changes. It may be associated with systemic fibrosis [4–6]. 

The disease is seen more in middle ages and is more common in women with a ratio of 4 to 1 in women to men (1).

The most important issue about Riedel's thyroiditis is its differential diagnoses including thyroid cancers such as thyroid follicular and Hurthle cell cancers which should be ruled out. Here we report two cases of Riedel's thyroiditis with two variable clinical presentations.

## 2. Case Presentation

The first case was a 51-year-old man who was referred to our clinic with a complaint of neck mass. The symptoms had begun 4–5 months ago (neck mass) and had been followed by face edema and muscle spasm. There was no history of any underlying disease or drug consumption. On thyroid examination, diffuse thyroid enlargement (about 60 gr) with firm density (woody) and uneven external surface was observed. Thyroid function test (T4 = 4.3, T3 = 92, TSH = 33, anti-TPO negative) showed hypothyroidism and other biochemical tests (including ESR, CBC, Ca, and P) were in normal limit. 

Thyroid radio isotope with TC99 revealed decreased and nonhomogenous uptake. Fine needle aspiration was performed, and the pathology report was adenomatous goiter with atypical cells. Levothyroxin 100 microgram/day was started.

By the presumptive diagnosis of thyroid cancer (anaplastic carcinoma) the patient underwent open wedge thyroid biopsy (of 2 lobes). 

Pathologic study showed dense fibrous tissue with neoplastic cells (probably undifferentiated thyroid carcinoma, small cell). 

Immunohistochemistry was done. In the immunohistochemistry study, EMA, cytokeratin, thyroglobulin, and calcitonin markers were negative and vimentin, LCA, CD20, and CD3 were positive. However, with review of FNA specimens and biopsy diagnosis of fibrosing thyroiditis was confirmed and malignancy was ruled out. 

Prednisolon 40 mg/day was started and has been tapered during three months and tomoxifen 20 mg/day. Neck pain improved and the thyroid size decreased from 60 gr to 30 gr. After 5 years of followup, now the patient is well and euthyroid with levothyroxine.

**Figure 1:**
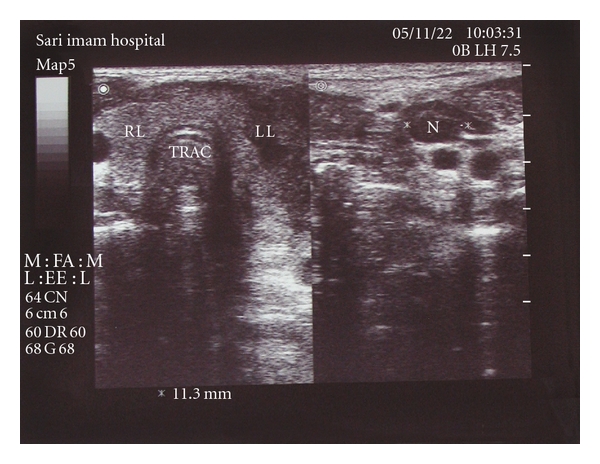
First sonography in the second case.

The second case was a 17-year-old man with the complaint of neck mass and a slight neck pain. The problem had begun one month ago and had progressed. The symptoms were followed by palpitation and sweating. On physical examination there was a neck mass in left side of thyroid and the right lobe was normal. Pulse rate was 100/min and there was a weak tremor. Neck ultrasound showed a heterogeneous solid neck mass in left thyroid lobe which had displaced neck vessels and sternocleidomastoid muscle. Also there were multiple neck lymphadenopathies in the anterior triangle of the same side, the diameter of the largest one was 12 mm (Figure 1).

**Figure 2:**
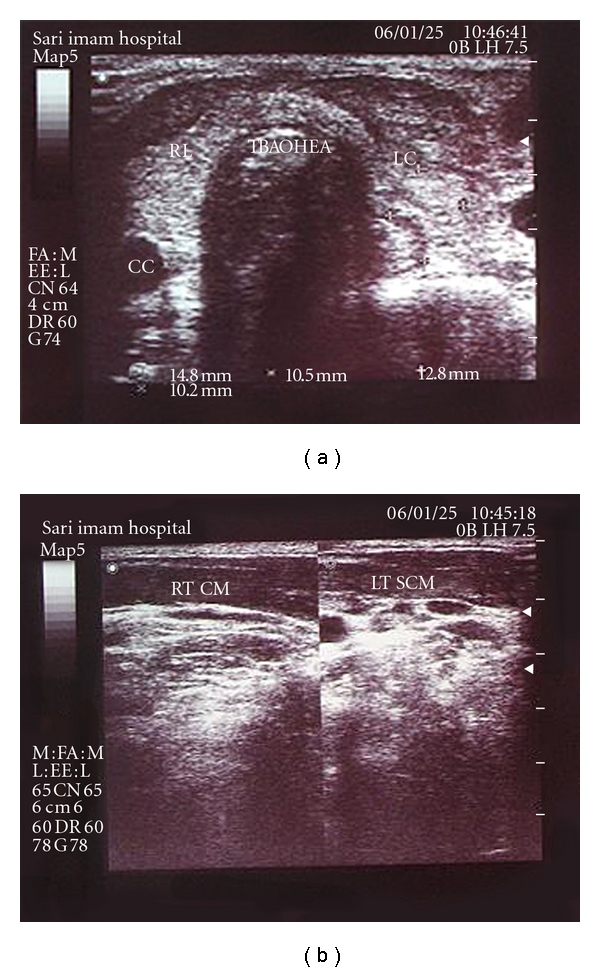
Second sonography findings in the second case.

**Figure 3:**
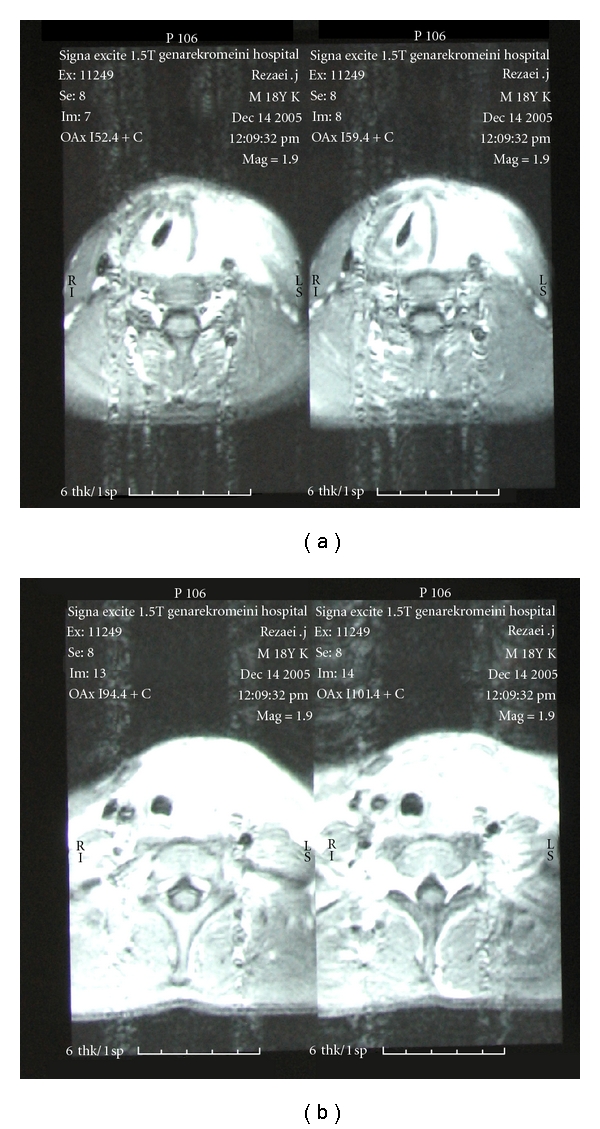
MRI findings in the second case.

Thyroid function test revealed thyrotoxicosis (T4 = 18.3 T3 = 265 TSH = less than 0.1). Anti-TPO was negative and ESR was elevated mildly (ESR = 38), and other biochemical tests (including, Ca, P, and CBC) were normal. Thyroid radio isotope with TC^99^ revealed decreased and nonhomogenous uptake thyroid biopsy showed an inflammatory process. In the second ultrasound, the mass size had been increased in comparison to the first one (Figure 2). Sonoguided biopsy showed infiltration of mononuclear cells and fibrosis. In neck MRI, a hypodense thyroid mass and involvement of surrounding soft tissue and left SCM muscle was found (Figure 3).

Abdomen and pelvic ultrasounds were normal. Open biopsy was performed, and, in direct observation, a white-to-gray mass with involvement of surrounding tissue and muscles was seen. Pathology exam showed infiltration of thyroid parenchyma by mononuclear cells, fibrosis, destruction, and involvement of SCM muscle.

Thyroid tissue was not seen and the diagnosis of Riedel's thyroiditis was confirmed. The patient was treated with propranolol 40 mg/two times/day and prednisolon 40 mg/day that was tapered during three months.

After one month of treatment the symptoms improved and neck mass size decreased, and in ultrasound examination SCM muscle was normal in two sides and left lobe was heterogenous and smaller than right lobe. TFT was in normal range.

## 3. Discussion

Riedel thyroiditis has been reported in middle ages in most of the cases and its process is gradual. Riedel's thyroiditis in our first case was in age 51 years but in second case it appeared in 17 year old. The disease process was slow in the first case but it was rapid in the second case. In most cases the disease is bilateral. In some cases one lobe is larger and rarely only one lobe is involved.

In case one, the disease was bilateral while in the second one only left lobe was involve. Most of the patients with Riedel's thyroiditis are euthyroid, but 30% of cases may have hypothyroidism or rarely thyrotoxicosis [3]. Our first case referred to us with hypothyroidism and the second one with thyrotoxicosis.

The disease is not associated with Pain but a mild Pain was present in neck region in our cases. The most important issue in diagnosis of Riedal's thyroiditis is its differential diagnoses with other diseases including Hashimoto thyroiditis and a form of papillary carcinoma which in both of them the severity of fibrosis is lesser than Riedel's thyroiditis and surrounding tissue is not involved. Another differential diagnosis is pauci cellular anaplastic carcinoma which in its pathology study in addition to fibrosis and necrosis, atypic cells, and atypic spindle cells will be seen, and epithelial markers are positive [7].

In MRI study malignant lesion is seen are hyperdense lesion, but Redial's thyroiditis is seen as hypodense ones [8].

In our two cases there was severe fibrosis with involvement of surrounding tissue and muscles. In thefirst case anaplastic carcinoma was the most probable diagnosis, which, in repeated studies, markers were negative, and in our second case there was lymphadenopathy which is very rare. The response to treatment was good in two cases especially in second one it was, very rapid response. Our study reports two cases with Rediel's thyroiditis and different symptoms in the 2 extremes of age, so it seems that age is a determining factor in presenting symptoms and signs, disease process, and response to treatment and should be considered in patient diagnosis and treatment. So, rare presentations such as unilateral involvement, thyrotoxicosis, high ESR, and lymphadenopathy were seen in our young patient.
